# ST-Segment Elevation Myocardial Infarction (STEMI) Secondary to Spontaneous Coronary Artery Dissection of the Right Coronary Artery: A Case Report

**DOI:** 10.7759/cureus.89792

**Published:** 2025-08-11

**Authors:** Dunya Alfaraj, Razan Alnimer, Bayan Ahmed, Fatima Abdali, Tahera Ali, Mohammad Alsharidah

**Affiliations:** 1 Department of Emergency, Imam Abdulrahman Bin Faisal University, King Fahd University Hospital, Dammam, SAU; 2 College of Medicine, Imam Abdulrahman Bin Faisal University, Dammam, SAU; 3 College of Medicine, Wenzhou Medical University, Wenzhou, CHN; 4 College of Medicine, Xi'an Jiaotong University, Xi'an, CHN

**Keywords:** acute coronary syndrome, case report, coronary angiography, fibromuscular dysplasia, intramural hematoma, spontaneous coronary artery dissection

## Abstract

Spontaneous coronary artery dissection (SCAD) is a rare and serious condition that can lead to acute coronary syndrome (ACS). It is characterized by a spontaneous tear in the coronary artery wall that is not related to trauma, medical intervention, or atherosclerotic disease, occurring particularly in young women and those in the peripartum period. Missed diagnoses are driven by a low suspicion of ACS in young women and a lack of clinician familiarity with the condition. Early recognition and appropriate management are key to improving patient outcomes. Recent studies suggest the improved recognition of SCAD due to dedicated SCAD registries and newer imaging techniques such as optical coherence tomography (OCT) and intravascular ultrasound (IVUS). We report a case of a 35-year-old woman who presented with retrosternal chest pain radiating to the left upper limb. Investigations were significant for an elevated troponin I level, while the initial electrocardiogram (ECG) was unremarkable. Coronary angiography revealed spiral dissection involving the right coronary artery, suggesting SCAD type 2. The patient was admitted as a case of SCAD for conservative treatment. During hospitalization, she developed a new episode of chest pain. Her repeated ECG revealed an inferior wall ST-segment elevation myocardial infarction (STEMI). She underwent percutaneous coronary intervention (PCI) with three overlapping drug-eluting stents. Furthermore, this article provides a detailed review of SCAD, including clinical presentation, associated conditions and factors, diagnosis, treatments, and complications based on literature synthesis.

## Introduction

Spontaneous coronary artery dissection (SCAD) is a rare condition. It is defined as a spontaneous separation of the coronary artery wall that is not related to trauma, medical intervention, or atherosclerotic disease. It is also known as the nonatherosclerotic SCAD. It is an unusual, important, and challenging cause of myocardial infarction (MI) in young women, especially outside the context of pregnancy and hereditary connective tissue disorders. Recent studies state SCAD as the cause of 35% of all acute coronary syndrome (ACS) events in women under the age of 50 years old. Young women, with no or few traditional cardiovascular risk factors, represent the “typical” SCAD patients. The true prevalence of SCAD remains uncertain, primarily because it is an underdiagnosed condition driven by a low suspicion of ACS in young women, even in the presence of classic presenting symptoms, and a lack of clinician familiarity with the condition. Clinical manifestations are widely variable, and it is similar to atherosclerotic ACS. Differentiating between SCAD and atherosclerotic disease is important as the management and investigation of SCAD differ. SCAD has two main theories that have been suggested, which are the “outside-in or media hemorrhage” and “inside-out or intima tear” mechanisms. Current literature favors the “outside-in” theory, proposing that intramural hematoma (IMH) develops sporadically within the tunica media, potentially from abnormalities within the vasa vasorum, causing a rupture into the true lumen [1-3].

## Case presentation

A 35-year-old woman, not known to have any medical illness, presented to the emergency department at King Fahd University Hospital in Al-Khobar, Saudi Arabia. She was complaining of retrosternal chest pain that started three hours ago. The pain was described as tightness radiating to the left upper limb with a 10 out of 10 in intensity. The pain was associated with shortness of breath, arm numbness, sweating, nausea, and vomiting. This was the first time that the patient had experienced such an event. She complained of similar mild chest pain for the past two weeks without any associated symptoms. The patient denied any history of fever, dizziness, headache, loss of consciousness, back pain, or weakness. She also denied a history of prolonged immobilization, recent travel, surgery, or trauma. Her vital signs were a temperature of 36.9°C, heart rate of 70/minute, respiratory rate of 20/minute, blood pressure of 140/72, and oxygen saturation of 100% on room air. The cardiovascular and pulmonary examinations were within normal limits, and there was no sign of heart failure (no S3 gallop and normal jugular venous pressure {JVP}). No abnormal sounds were heard upon chest auscultation, and no murmurs were appreciated.

**Figure 1:**
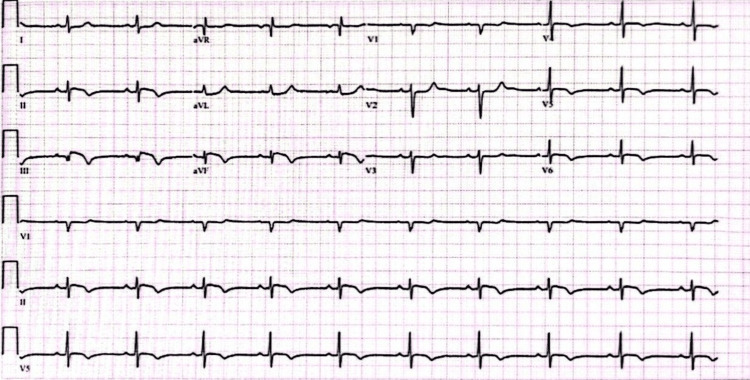
ECG showing inferior wall STEMI ECG, electrocardiogram; STEMI, ST-segment elevation myocardial infarction

The patient was administered aspirin and underwent laboratory and radiological investigations. Laboratory results were significant for troponin I of 2.909 ng/mL (<0.04 ng/mL for healthy individuals and <1.5 ng/mL for non-acute MI), while the initial electrocardiogram (ECG) was unremarkable. Echocardiography showed inferior and inferolateral hypokinesia with a normal ejection fraction. In coronary angiography, it showed spiral dissection involving the right coronary artery with a lack of plaque rupture, suggesting SCAD type 2. The patient was admitted as a case of SCAD for conservative treatment. During hospitalization, the patient developed a new episode of chest pain. Her repeated ECG revealed an inferior wall ST-segment elevation myocardial infarction (STEMI) (Figure 1). Thus, the cath lab was activated, where she underwent percutaneous coronary intervention (PCI) with three overlapping drug-eluting stents. Further evaluation and investigations were done and ruled out systemic vasculitis. Laboratory results were negative for antineutrophil cytoplasmic antibody (ANCA), Sjögren’s syndrome antigen A (SSA), Sjögren’s syndrome antigen B (SSB), anti-double-stranded deoxyribonucleic acid (dsDNA), and anti-Smith antibodies. An iron profile was done due to low mean corpuscular volume (MCV). According to the results of the iron profile, the patient was diagnosed with iron deficiency anemia. See Table 1 for detailed laboratory results. The patient’s condition improved, and the patient had an uneventful hospital stay after PCI without any complications. She was discharged on aspirin, ticagrelor, bisoprolol, atorvastatin, and lisinopril. She was also advised to avoid any stressful events and apply lifestyle modifications such as cardiac rehabilitation, nutritional counseling, physical activity counseling, and psychosocial management.

**Table 1:** Laboratory results at the time of admission WBC, white blood cells; RBC, red blood cells; Hgb, hemoglobin; Hct, hematocrit; MCV, mean corpuscular volume; MCH, mean corpuscular hemoglobin; MCHC, mean corpuscular hemoglobin concentration; RDW, red cell distribution width; MPV, mean platelet volume; BUN, blood urea nitrogen; AST, aspartate aminotransferase; ALT, alanine aminotransferase; LDH, lactate dehydrogenase; GGTP, gamma-glutamyl transpeptidase; MI, myocardial infarction

Laboratory parameters	Value	Normal value
Complete blood count		
WBC	8.3 K/µL	4-11 K/µL
RBC	5.03 million cells/µL	4.2-5.5 million cells/µL
Hgb	11.8 g/dL	12-16 g/dL
Hct	36.8%	37%-47%
MCV	73.3 fL	80-94 fL
MCH	23.4 pg	27-32 pg
MCHC	32.0 g/dL	32-36 g/dL
RDW	14.7%	11.5%-14.5%
Platelets	223 K/µL	140-450 K/µL
MPV	13.1 fL	7.2-11.1 fL
Neutrophils	81.2%, 6.7 K/µL	40%-75%, 2-7.5 K/µL
Lymphocytes	13.4%, 1.1 K/µL	20%-45%, 1-5 K/µL
Monocytes	4%, 0.3 K/µL	3%-9%, 0.11-1 K/µL
Eosinophils	0.3%, 0 K/µL	0%-6%, 0-0.8 K/µL
Basophils	0.3%, 0 K/µL	0%, 0-0.02 K/µL
Renal function test		
BUN	9 mg/dL	7-18.7 mg/dL
Creatinine	0.73 mg/dL	0.6-1.3 mg/dL
Sodium	139 mEq/L	136-145 mEq/L
Potassium	4.9 mEq/L	3.5-5.1 mEq/L
Chloride	106 mEq/L	98-107 mEq/L
Liver function test		
Total bilirubin	0.9 mg/dL	0.2-1.2 mg/dL
Direct bilirubin	0.3 mg/dL	0.1-0.5 mg/dL
Total Protein	7.4 g/dL	6.4-8.3 g/dL
Albumin	4.4 g/dL	3.2-5.2 g/dL
Alkaline phosphatase	45 U/L	40-150 U/L
AST	17 U/L	5-34 U/L
ALT	10 U/L	5-55 U/L
LDH	173 U/L	125-220 U/L
GGTP	8 U/L	9-36 U/L
Other laboratory parameters		
Troponin I	2.909 ng/mL	<0.04 ng/mL for healthy individuals and <1.5 ng/mL for non-acute MI
Random glucose	103 mg/dL	70-140 mg/dL
Lipase	22 U/L	8-78 U/L

## Discussion

Clinical presentation

Despite the wide range of clinical presentations and severities of SCAD, most patients present with ACS and the elevation of cardiac enzymes; 26%-87% present with STEMI, 13%-69% present with non-ST-segment elevation myocardial infarction (NSTEMI), 3%-11% present with sudden cardiac death or ventricular arrhythmias, and nearly 2%-5% of patients present with cardiogenic shock. The presetting symptoms are consistent with atherosclerotic ACS, with chest pain being the most predominant as reported in around 96% of patients, as shown in Table 2 [2-4]. Ventricular arrhythmias can occur early at the time of presentation or later during hospitalization. It is influenced by factors such as ischemia, reperfusion injury, and left ventricular (LV) ejection fraction [2,4,5].

**Table 2:** Frequency of presenting symptoms of spontaneous coronary artery dissection Data has been sourced from Hayes et al. [3]

Symptoms	Frequency
Chest pain	95.9%
Radiation to the arm	51.5%
Nausea or vomiting	23.7%
Radiation to the neck	22.2%
Diaphoresis	21.1%
Dyspnea	19.6%
Back pain	13.9%
Dizziness	8.8%
Ventricular tachycardia or ventricular fibrillation	7.2%
Fatigue	5.2%
Headache	1.5%
Syncope	0.5%

Associated conditions and factors

The underlying etiology of SCAD is suggested to be multifactorial. It occurs in patients who have a vulnerable arterial substrate with an accompanying trigger. These triggers include precipitating physical or psychological stressors, the use of illicit substances, underlying arteriopathies, or hormonal factors such as pregnancy [2,6]. See Table 3 for conditions and factors associated with SCAD.

**Table 3:** Conditions and factors associated with spontaneous coronary artery dissection Data has been sourced from Hayes et al. [3] β-hCG: beta-human chorionic gonadotropin

Conditions and factors
Fibromuscular dysplasia
Pregnancy
Multiparity (≥4 births)
Inherited arteriopathy and connective tissue disorder
Marfan syndrome, Loeys-Dietz syndrome, vascular Ehlers-Danlos syndrome, alpha-1 antitrypsin deficiency, and polycystic kidney disease
Exogenous hormones
Oral contraceptives, postmenopausal therapy, infertility treatments, testosterone, and corticosteroids
Systemic inflammatory disease
Systemic lupus erythematosus, Crohn disease, ulcerative colitis, polyarteritis nodosa, sarcoidosis, Churg-Strauss syndrome, Wegener granulomatosis, rheumatoid arthritis, Kawasaki disease, and celiac disease
Migraine headache
Precipitating factors: intense exercise (isometric or aerobic), intense Valsalva, retching, vomiting, bowel movement, coughing, lifting heavy objects, intense emotional stress, labor and delivery, recreational drugs (cocaine and methamphetamines), exogenous hormones/hormone modulators, β-hCG injections, corticosteroid injections, and clomiphene

The most reported precipitants are emotional stressors in women and extreme physical stress such as intense isometric exercise and weightlifting, which is often reported in men. Catecholamine has been shown to surge during stressful events and lead to shear stress in the coronary artery wall, which may contribute to the pathophysiology of SCAD. Although this hypothesis has not been specifically tested in patients with SCAD, a similar mechanism was proposed in other stress-induced cardiovascular conditions such as Takotsubo syndrome [3].

Fibromuscular dysplasia (FMD) is the most commonly associated condition reported with SCAD, as it can lead to the development of arterial aneurysms and dissections in some patients [6,7]. It is recommended that all patients with SCAD perform a one-time radiological test for all blood vessels to detect any abnormal arterial findings or FMD lesions, which can impact both management and prognosis [8].

SCAD is observed to be the most common cause of pregnancy-related MI, mostly in the third trimester or during the early postpartum period. Hormonal changes in each pregnancy may lead to the chronic repetitive impairment of arterial wall integrity, which happens simultaneously with the hemodynamic changes in pregnancy, such as increased cardiac output. Several studies have reported that the risk of SCAD is higher in women who have had multiple pregnancies in a threshold-based relationship rather than a linear. In addition, women who use a hormonal replacement therapy or with a high-risk pregnancy, such as preeclampsia and gestational diabetes, have a higher risk of SCAD [1,2,9,10].

Diagnosis

For diagnosis, A 12-lead ECG is the initial diagnostic test. ECG abnormalities suggestive of ischemia are frequently seen in patients with SCAD at presentation. Rarely, a normal ECG is also seen at presentation. In addition, echocardiography is routinely used to observe abnormalities in the focal wall motion, which can aid in defining possible sites of dissection. Unremarkable findings in echocardiography do not rule out SCAD, particularly in cases of early SCAD or cases of small or subendocardial infarctions. The overall LV ejection fraction is often preserved in SCAD. However, it can lead to reduced LV ejection fraction in severe cases. Young women, with no or few traditional cardiovascular risk factors, represent the “typical” SCAD patients, making this condition underdiagnosed. Nevertheless, there has been an increase in cases of SCAD in recent years that is apparently due to the increased use of coronary angiography, which should be done as early as possible to confirm the diagnosis in most patients with SCAD and no history of trauma. SCAD is classified into three main types depending on the coronary angiographic appearance (Table 4). Only one coronary artery is affected in most cases, which is usually the left anterior descending (LAD) coronary artery. Our patient had a right coronary artery involvement, which makes this case even more rare. She underwent coronary angiography, and it revealed a spiral dissection of the right coronary artery. Thus, this case is classified as SCAD type 2, which is the most commonly observed. However, angiography may sometimes fail to detect changes of intimal disruption, leading to the failure of diagnosis. In this case, advanced imaging, such as optical coherence tomography (OCT) or intravascular ultrasound (IVUS), can provide more detailed information to detect IMH and/or double lumen [1-4,11,12]. They are often required for type 2 and 3 SCAD. Comparative investigations between IVUS and OCT showed that OCT was more sensitive and more effective than IVUS at detecting more SCAD features, including intimal tears and flaps [13,14].

**Table 4:** Spontaneous coronary artery dissection types based on the coronary angiographic appearance

Type	Description
Type 1	Multiple radiolucent lumens or contrast staining of the arterial wall. The classic type with pathognomonic appearance [2,4]
Type 2	Diffuse stenosis of variable length and severity with the abrupt change in the arterial caliber from the normal diameter to diffuse smooth narrowing. The most common type [2,4,14]
Type 3	Focal or tubular stenosis (typically <20 mm), which requires intracoronary imaging to confirm the diagnosis. This type mimics atherosclerosis [2,4]

Management

Conservative management is initially attempted in stable patients. Over 90% of these patients have demonstrated angiographic healing, usually within a month. However, over half of them return within the first week. The medical management of ACS due to SCAD differs from atherosclerotic ACS. In atherosclerotic ACS, patients get started on dual antiplatelets (DAPT) and anticoagulation. In SCAD, on one hand, anticoagulation should be stopped once SCAD has been identified by angiography, as it can worsen IMH. On the other hand, there is no evidence to support the use of DAPT in SCAD. However, the expert consensus advises long-term aspirin use. DAPT, including aspirin and clopidogrel, can be used in SCAD in cases of stent and high-risk features such as thrombus burden, critical stenosis, and decreased coronary flow. Strong P2Y12 inhibitors, such as ticagrelor and prasugrel, should be avoided in SCAD due to the increased risk of bleeding. Compared to clopidogrel, ticagrelor has more advantages in case of high-risk ischemic episodes. Furthermore, thrombolytic therapy is not used in acute SCAD due to the risk of cardiac tamponade and coronary artery rupture. Beta-blockers are recommended as they reduce the risk of recurrent SCAD.

Moreover, chest pain should be managed with antianginal medications to reduce the frequency of hospital stays. These patients should also be advised to avoid prolonged Valsalva maneuvers, high-intensity activity, and isometric exercise. PCI is performed in patients with persistent ischemia or hemodynamic instability. The downside of the PCI procedure is that most of the SCAD lesions are less responsive to PCI due to their distant position, leading to higher risks of propagation and complications. Serious PCI complications involve aorto-ostial dissection, reduced flow in proximal coronary vessels, or unplanned left mainstem stenting or coronary artery bypass grafting (CABG). Patients may undergo CABG in case of refractory PCI, refractory conservative treatment, refractory ischemia, proximal dissections, or dissections in the left main coronary artery. However, there is a high rate of graft failure in the long-term (graft patency of 27%) due to graft occlusion caused by the competing flow in the healed native vessels. Follow-up can be done with coronary computed tomography angiography (CCTA) or repeated invasive coronary angiography. CCTA is often preferred due to its noninvasiveness, while repeat angiography is only done for patients who have a positive functional study and high-risk anatomy. Cardiac rehabilitation is highly recommended. It has shown evidence-based improvement in measures of psychosocial and physical aspects, metabolic values, and reduced frequency of symptoms [2,3,15-18].

Complications

The complications of SCAD include congestive heart failure, cardiogenic shock, ventricular tachyarrhythmias, and ventricular free wall or septal rupture. In the outpatient setting, chest pain following SCAD is frequently reported. Recurrence is common, occurring in about one out of every five patients. It is defined as a new dissection away from the original site. Some factors have been linked with recurrence, such as connective tissue disorder, genetically mediated arterial disease, coronary tortuosity, history of migraine headache, FMD, hypertension, pregnancy, and hormonal therapy. In addition, these patients are susceptible to psychological stress, anxiety, or depression. Patients should be screened prior to discharge for mental health conditions such as post-traumatic stress disorder, depression, anxiety, and reduced quality of life. Patient Health Questionnaire-2 (PHQ-2) can be used for initial evaluation (depressed mood and anhedonia) and then the more advanced PHQ-9 (Table 5) accordingly, which has been shown to have reasonable sensitivity and specificity for patients with coronary heart disease [7,15,19,20]. SCAD can be intimidating to patients due to its unfamiliarity and uncommon nature. Thus, numerous community support groups have been formed for psychological benefit and support from other patients [3].

**Table 5:** Patient Health Questionnaire-9 (PHQ-9) Data has been sourced from Williams et al. [20]

Over the last two weeks, how often have you been bothered by any of the following problems?				
	Not at all	Several days	More than half the days	Nearly every day
Little interest or pleasure in doing things	0	1	2	3
Feeling down, depressed, or hopeless	0	1	2	3
Trouble falling or staying asleep or sleeping too much	0	1	2	3
Feeling tired or having little energy	0	1	2	3
Poor appetite or overeating	0	1	2	3
Feeling bad about yourself or that you are a failure or have let yourself or your family down	0	1	2	3
Trouble concentrating on things, such as reading the newspaper or watching television	0	1	2	3
Moving or speaking so slowly that other people could have noticed or the opposite, being so fidgety or restless that you have been moving around a lot more than usual	0	1	2	3
Thoughts that you would be better off dead or of hurting yourself in some way	0	1	2	3

## Conclusions

SCAD is a rare and potentially life-threatening condition, particularly in young women and those in the peripartum period. Its diagnosis can be challenging as it often presents similarly to other causes of ACS and due to low suspicion and clinical unfamiliarity with such a condition in young women. Advances in imaging techniques have significantly improved the ability to recognize SCAD. Conservative management is the mainstay of treatment for stable patients, and interventional treatments are reserved for high-risk or unstable patients. Early recognition and tailored treatment plans are crucial for improving patient outcomes. Ongoing research is needed to better understand this condition and refine different treatment approaches.
